# European stakeholders’ perspectives on implementation potential of precision weed control: the case of autonomous vehicles with laser treatment

**DOI:** 10.1007/s11119-023-10037-5

**Published:** 2023-06-12

**Authors:** Duc Tran, Joachim J. Schouteten, Margo Degieter, Janusz Krupanek, Wanda Jarosz, Alvaro Areta, Luis Emmi, Hans De Steur, Xavier Gellynck

**Affiliations:** 1 Department of Agricultural Economics, Ghent University, 653 Coupure Links, 9000 Ghent, Belgium; 2 Instytut Ekologii Terenów Uprzemysłowionych (IETU), Dział Badań i Rozwoju, Katowice, Poland; 3 Spanish coordinator of Farmers and Livestock Breeders (COAG), Agustín de Bethancourt, 17 5ª, 28003 Madrid, Spain; 4 Centre for Automation and Robotics (UPM-CSIC), 28500 Arganda del Rey, Madrid, Spain

**Keywords:** Agricultural robots, Weed control, Laser, SWOT, PESTLE, Market potential, Stakeholders’ perception

## Abstract

Weed control is a basic agricultural practice, typically achieved through herbicides and mechanical weeders. Because of the negative environmental impacts of these tools, alternative solutions are being developed and adopted worldwide. Following recent technical developments, an autonomous laser-based weeding system (ALWS) now offers a possible solution for sustainable weed control. However, beyond recent proof of performance, little is known about the adoption potential of such a system. This study assesses the adoption potential of ALWS, using a mixed-method approach. First, six macro-environmental factors regarding the adoption of ALWS were determined. This assessment is referred to as a Political, Economic, Social, Technological, Legal, Environmental (PESTLE) analysis and is conducted in a form of a literature review initiated by expert consultations. Second, a range of European stakeholders’ perceptions of ALWS was evaluated in four focus-group discussions (n = 55), using a strengths, weaknesses, opportunities, threats (SWOT) analysis. The factors identified in the PESTLE and SWOT analyses were subsequently merged to provide a comprehensive overview of the adoption potential of ALWS. Labour reduction, precision treatment and environmental sustainability were found to be the most important advantages of ALWS. High costs and performance uncertainty were identified as the main weaknesses. To promote the adoption of ALWS, this study recommends the following: (1) Concrete performance results, both technical and economic, should be communicated to farmers. (2) Farmers’ knowledge of precision agriculture should be improved. (3) Advantage should be taken of policies that are favourable towards non-chemical methods and the high demand for organic products. This article also extensively discusses regulatory barriers, the risks posed to the safety of both humans and the machines involved, technological challenges and requirements, and policy recommendations related to ALWS adoption.

## Introduction

Weed growth is a major factor in the reduction of crop yield, therefore weed control has always been a crucial aspect of crop cultivation. Herbicides are the most common method of weed control (Young & Pierce, 2014), but such chemicals have serious negative impacts, such as the increasing emergence of herbicide-resistant weeds and the presence of toxic residues in agricultural ecosystems (Desquilbet et al., 2019). Because of the harmful impacts of herbicides on the environment and human health, several countries have stringent regulations regarding the use of agricultural chemicals (Petit et al., 2015; Westwood et al., 2018). The growing demand for organic food products simultaneously motivates the reduced use of synthetic herbicides in agriculture.

In organic farming, soil tillage and crop rotation are the main alternatives to herbicides (Cloutier & Leblanc, 2001; Liebman & Dyck, 1993). These approaches reduce chemical usage and prevent toxic residues. Mechanical tools can assist with soil tillage, reducing the production cost of manual weed control. However, soil-tillage machinery continue to have drawbacks. Rabier et al. (2017) indicated that mechanical weeders are less effective than herbicides because common mechanical weeders (e.g., hoeing, rotating blades) cannot target in-row weeds. Furthermore, soil disturbance due to tillage can harm beneficial soil organisms, such as earthworms, and cause soil erosion and the leaching of plant nutrients (Chatterjee & Lal, 2009). In addition, heavy machinery, such as tractors, causes soil compaction. Such compaction lowers the levels of oxygen in the soil required for root respiration, triggers weed germination and destroys the habitat of soil-dwelling animals.

Concerns regarding current approaches to weed control demand innovative solutions, of which laser-based weed control is one. A laser beam is created by stimulated emissions of electromagnetic radiation via optical amplification (Andreasen et al., 2022). Laser-based treatment is classified as a physical weed-control method (Young & Pierce, 2014). Several technical studies have examined the potential use of laser beams in weed control. Heisel et al. (2001) found that a laser beam can cut weed stems and avoid the regrowth of dicotyledonous plants if the beam cuts below the meristems of such plants. Mathiassen et al. (2006) examined different determinants of the effectiveness of laser weed control. They found wavelength, exposure time, laser power and spot size to be crucial. However, the effectiveness of laser weeding can vary among weed species.

To selectively kill weeds, a laser-based weed control system requires the support of recognition systems (Wang et al., 2019a). Such recognition systems use artificial intelligence (AI) to distinguish weeds from crop plants and eradicate weeds in a selective manner. This feature allows laser-based weed control to fit into the scope of precision agriculture (Christensen et al., 2009). Marx et al. (2012) introduced the laser irradiation model for weed control but managed to detect only one weed type in laboratory conditions. Xiong et al. (2017) developed a prototype robot that can detect weeds in indoor environments and direct laser beams to eradicate the weeds. Rakhmatulin and Andreasen (2020) investigated the impact of different laser-beam strengths on weeds and found that 5-watt laser beams killed weed plants efficiently. However, beams of this strength can also damage crops if they are split during the weeding process.

In addition to recognition systems, autonomous vehicles offer other benefits that make them suitable for laser-based weed control. Since their movement can be programmed and monitored (Slaughter et al., 2008), autonomous vehicles give recognition systems sufficient time to detect weeds and activate laser treatment. Furthermore, autonomous vehicles can theoretically work 24/7, which maximises weeding capacity. Consequently, autonomous vehicles and robots have gained popularity among stakeholders in agriculture. Von Veltheim and Heise (2021) found that German farmers had a positive attitude towards autonomous field robots. In their Delphi study, Ammann et al. (2022) found that agricultural experts in Switzerland considered robots and autonomous machines to be the second-most-promising technology for precision agriculture. Similarly, 22.6% of surveyed farmers in Germany stated that they planned to adopt field-crop robots in the next 5 years (Spykman et al., 2021).

In 2021, the first model of an autonomous laser-based weeding system (ALWS) became commercially available in the United States (Manning, 2022). In Europe, several initiatives aim to develop and advance similar systems (Andreasen, et al., 2022). While technical studies on agricultural robots, such as ALWS, are relatively abundant, studies on the socio-economic aspects of such technologies remain scarce (Lowenberg-DeBoer et al., 2020; Pathak et al., 2019). The success factors of the commercialisation of agricultural robots and their wide-scale adoption remain unclear (Pathak et al., 2019; Reichardt et al., 2009). Particularly in the case of ALWS, farmers may find the change from conventional weed-control practices using herbicides and soil tillage to laser-based methods to be a challenge. Macro-environmental factors, such as existing policies, legislation and market competition, can also hinder adoption. Furthermore, because of the novelty of agricultural robots and ALWS, little is known about stakeholders’ perceptions of their potential implementation.

To address this lack of knowledge, this study examines the adoption potential of ALWS. First, a literature review that focuses on political, economic, social, technological, legal and environmental (PESTLE) factors provides a comprehensive overview of the business environment in which the adoption of ALWS would occur. Second, a SWOT analysis examines stakeholders’ perceptions of ALWS adoption and identifies the most important factors for the strategic implementation of ALWS. Finally, the factors identified in the literature review and SWOT analysis are compared to (1) identify the gaps in the literature, (2) put the findings in literature into practitioners’ perspectives, and (3) highlight the issues in the literature that stakeholders might have been uninformed.

Since the characteristics of the agricultural machinery market and the policy milieu vary greatly between countries, the European agricultural market was selected as a sample that would ensure a sufficiently detailed analysis. Europe was chosen for the following reasons: (1) While a fully commercial ALWS has not become available in Europe, the development of such a system in a number of European Union (EU) projects may bring it to the market in the foreseeable future. (2) Laser-based systems correspond with the EU’s stated ambition to make agriculture sustainable (Ulmann, 2020). (3) The high cost of agricultural labour in Europe makes the adoption of automated solutions, such as ALWS, more relevant than in developing countries, where low-skilled labour is cheaper (Farm Europe, 2021).

## Materials and methods

**Fig. 1:**
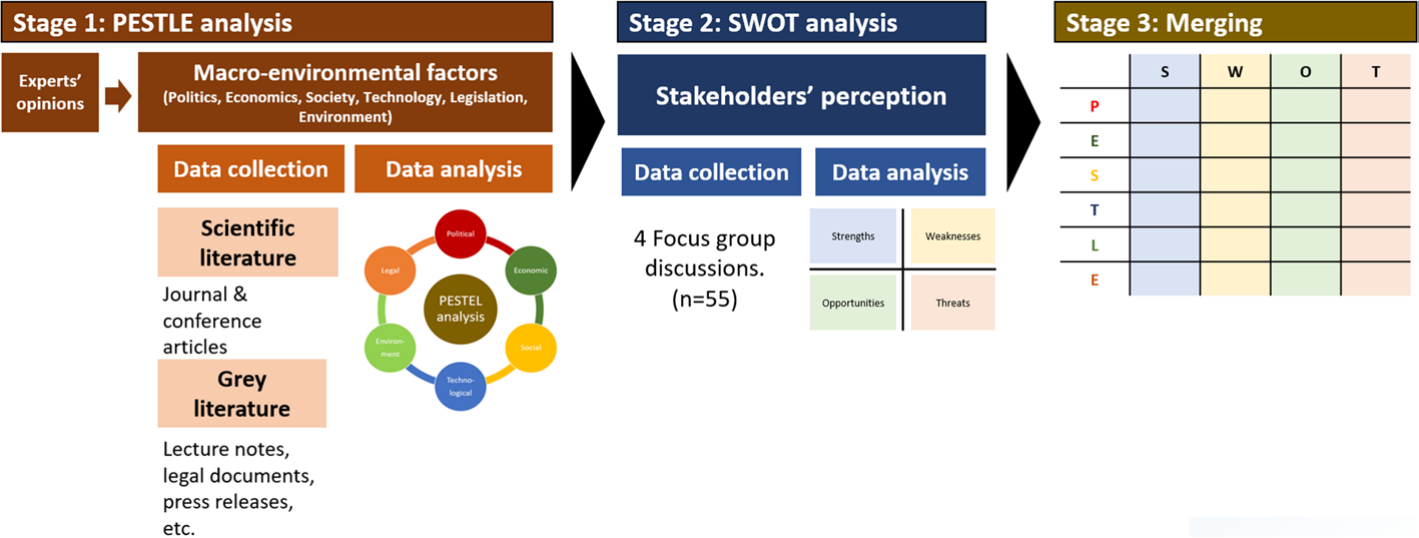
Three stages of data collection and analysis

This study followed a mixed-method approach to investigate the adoption potential of ALWS, in three stages (Fig. 1).

In the first stage, the political, economic, social, technological, legal and environmental factors that would impact the adoption of ALWS were identified. The assessment of these six macro-environmental factors in a business is referred to as a PESTLE analysis (Perera, 2017). A PESTLE analysis provides a comprehensive overview of the current business environment, which helps business managers and industry leaders make long-term business plans and orientate their organisations (Kremer & Symmons, 2015). In this study, the PESTLE assessment was conducted via a literature review to provide a factual overview of the potential of the adoption of ALWS. The literature review was initiated by consultations with experts. In the second stage, stakeholders’ perceptions of the adoption of ALWS were gathered in four focus-group discussions, followed by a SWOT analysis. SWOT analyses have often been used in studies that aim to capture stakeholders’ perceptions of innovations (Olum et al., 2018; Rutsaert et al., 2014) because of its ease of use and popularity among participants. While the PESTLE analysis provides a broad picture of the business environment regarding the adoption of ALWS, the SWOT analysis highlights the most important factors from the stakeholders’ perspective, which might help machinery producers to map out their business strategies. Lastly, the factors found in the PESTLE and SWOT analysis were merged and classified into each other categories to provide a comprehensive picture of the adoption potential of ALWS.

### PESTLE analysis

To initiate the PESTLE analysis, six experts were invited to consult the literature review on the adoption of ALWS (Fig. 1). The experts were recruited based on their relevant expertise, which corresponded with the six dimensions of the PESTLE analysis (Table 1). Each expert highlighted the key issues in the adoption of ALWS. These issues corresponded with the PESTLE framework and were used to guide the literature review phase. A wide range of sources was consulted for the review: Both scientific literature and grey literature, such as lecture notes, legal documents and press releases, were included.

**Table 1:** Description of the six experts in Stage 1

ID	Dimensions	Occupation/Title
1	Politics	Board member of IFOAM Organics Europe, chair of the board of directors of an organic cooperative
2	Economic, Society	Professor in agribusiness economics
3	Technology	Agricultural technology developer, project manager
4	Legislation	Professor in agricultural law
5	Environment	Specialist in life cycle assessment for industrial areas
6	Environment	Professor in plant and environmental sciences

### Focus group discussions

In the second stage, four focus-group discussions were arranged with a wide range of stakeholders to examine their perceptions of the application potential of ALWS. The four groups followed a uniform procedure, but the languages of communication varied depending on the participants. The first focus group served as a pilot and was conducted in English. This group involved stakeholders with international experience from Belgium, Denmark, Finland, Germany, Italy, Poland, Spain and Switzerland. Based on the pilot, three country-specific focus-group discussions were arranged in Belgium/Netherlands, Poland and Spain. This meant that the three main European regions—Western, Eastern and Southern Europe were covered (Hobbs, 2021). These national focus-group discussions aimed to explore stakeholders’ perspectives in country-specific contexts and were conducted in the corresponding national languages, namely Dutch, Polish and Spanish.

These focus-group discussions were conducted virtually (from December 2021 to February 2022) due to the COVID-19 pandemic. In total, 55 participants attended, and no participant attended more than one discussion. Table 2 shows the number of participants in each focus-group discussion. Personal data of the participants were processed to ensure anonymity.

**Table 2:** Participants in focus groups

Focus group (by languages of discussion)	English	Dutch	Polish	Spanish	Total
Farmers and representatives of agricultural cooperatives	7	7	2	5	21
Developers, providers of and dealers in machinery	1	3	1	3	8
Researchers	5	3	2	1	11
Advisory bodies and/or policymakers	2	1	11	2	15
Total	15	13	16	11	55

### Focus-group procedure and SWOT analysis

**Fig. 2:**
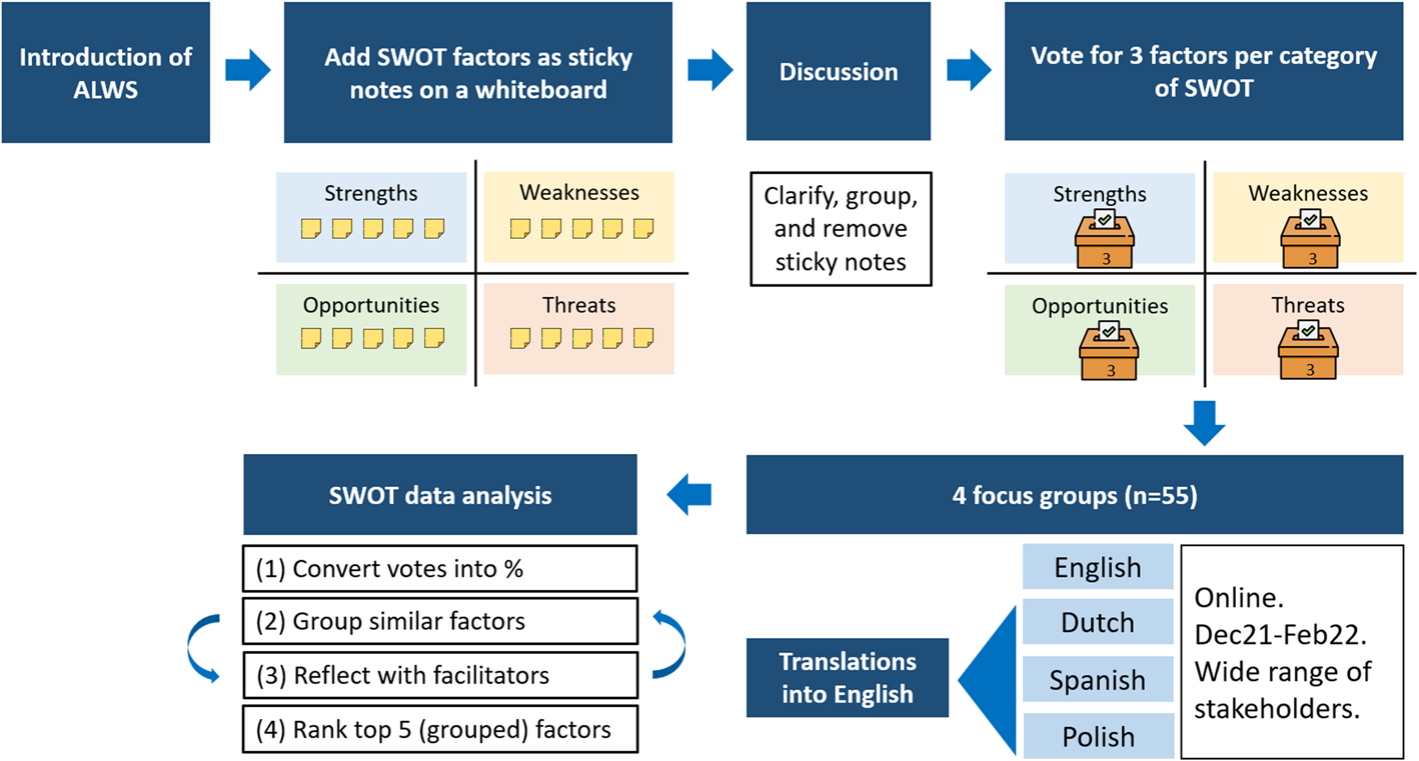
Procedure of the focus-group discussions

In each focus-group discussion, participants were first given a brief description of the main configuration of an ALWS prototype, consisting of four components: (1) a laser treatment system, (2) a weed-crop recognition system, (3) an autonomous vehicle, and (4) a smart central control. After this introduction, participants were asked to write down the factors related to the strengths, weaknesses, opportunities and threats (SWOT) of the adoption of ALWS on virtual sticky notes (Fig. 2). Each factor was written on a separate sticky note. This was followed by a discussion round to clarify the meaning of the factors that had been noted. Duplicate notes were either merged or removed. Finally, participants were asked to vote anonymously for three factors/sticky notes per SWOT category that they regarded as the most important. The content of the sticky notes and their corresponding votes were recorded for subsequent analysis.

Except for the first discussion, which was in English, the SWOT data of each focus group were translated into English. Since the groups varied in size, the number of votes for each factor was not directly comparable: The group with more participants would have factors that received more votes. To neutralise the difference, the number of votes was converted into a percentage of the number of participants per workshop. After standardising the voting scores, the factors in each SWOT category were grouped based on their content. The group facilitators were asked to validate the new groupings to avoid misinterpretation and biases. Next, the five (grouped) factors that received the highest percentage of votes in each SWOT category were selected for further discussion in a SWOT analysis.

## Results and discussion

### PESTLE analysis

#### Political factors

The use of agricultural chemicals, including pesticides and herbicides, is heavily regulated in the EU (Bonanno et al., 2017; Kudsk & Mathiassen, 2020). Directive 2009/128/EC aims to achieve the sustainable use of agricultural chemicals in the EU by promoting Integrated Pest Management and alternative approaches or techniques, such as non-chemical alternatives to pesticides (European Commission, 2021a).

The Green Deal announced by the EU in 2020 has outlined ambitious goals for the agricultural sector (Helga et al., 2022). In terms of this deal, the EU aims to reduce the use and risk of chemical pesticides by 50% and lower the resultant nutrient losses by at least 50%. A further goal is for 25% of agricultural land to be under organic farming by 2030. The EU Action Plan for Organic Agriculture, which is contained in the Green Deal, emphasises the importance of finding alternatives to contentious chemical inputs by accessing funding provided by Horizon European, the EU’s key funding programme (European Commission, 2021b). In addition, the European Commission has focused on reforming the common agricultural policy (CAP) to make it more compatible with the Green Deal. The updated version of CAP emphasises local conditions and needs and adopts a more flexible and results-based approach to achieve the EU’s sustainability goals (European Commission, 2020). In particular, CAP allocates 25% of the budget for direct payments to eco-schemes, thus providing sufficient incentives for climate- and eco-friendly practices and approaches.

Regulations that are unfavourable towards chemical weed control pave the way for the adoption of alternative solutions, of which ALWS is one. Furthermore, incentives (e.g., funding) for organic farming and other sustainable practices in Europe can be trickled down to the development of ALWS. In fact, a few projects funded by the public and private sector, namely WeLASER and Weedbot, are already developing ALWS within the EU.

#### Economic factors

ALWS can be of particular value in organic farming as it eliminates the need for chemical pesticides and significantly reduces manual weed control, which is normally a feature of organic farming. In the EU, the market for organic products is booming: Organic retail sales reached €44.8 billion in 2020 (equivalent to a 15.1% year-on-year increase) allowing farmers to add value to their products (Helga et al., 2022). Nevertheless, organic farmland expansion in 2020 increased by only 5.3% compared to 2019, which indicates that the growth of the organic market exceeds the expansion of organic farmland (Helga et al., 2022). Even though the average increase in organic agricultural land in the EU was about 65% from 2009 to 2019, EU member states need to make greater progress to achieve the Green Deal goal of 25% of farmland being organic by 2030. At present, only Austria has achieved this target. Furthermore, EU consumers favour products that are labelled as organic and are willing to pay a premium for such products (Janssen & Hamm, 2012; Schouteten, et al., 2019). Given the ever-increasing demand for organic products and the expansion of organic farmland in the EU, the prospects for sustainable weed control approaches such as ALWS are promising.

As mentioned above, ALWS can eliminate the need for manual weed control in organic farming. Depending on whether hand weeding is used or not, weed-control costs and related investment in machinery in the EU can vary from €50 to €1 500 per ha per year (European Parliamentary Research Service, 2021). Given the high cost of farm labour in the EU (Farm Europe, 2021), autonomous systems, such as ALWS, have the potential to reduce production costs in the long term.

Supply-chain disruptions can negatively impact the development of new technologies, such as ALWS. For example, COVID-19 lockdown measures caused shipping delays and skyrocketing shipping costs, which eventually disrupted the global supply chain (Barrett, 2021). Such disruption has a domino effect. A case in point is the shortage of semiconductors, which has halted production in several technological industries (Baraniuk, 2021). Similarly, the developers and manufacturers of machinery may face difficulties in importing the necessary electronic components for ALWS.

Furthermore, ALWS is a high consumer of energy because it performs several functions, including laser treatment, weed recognition and mobility tasks. Hence, the energy crisis in the EU, which has been exacerbated by the Russia-Ukraine conflict, can have a negative impact on the operation costs of ALWS (Dahm, 2022). If ALWS were not dependent on fossil fuels, the impact of the energy crisis could be reduced. However, renewable energy options, such as solar panels, may be insufficient to provide electrical power for high-performance industrial machines such as ALWS.

Ultimately, however, as is the case with similar precision agriculture techniques, the adoption of ALWS may be hindered by the high initial investment cost, as has been stated in several previous studies (Pathak et al., 2019; Reichardt et al., 2009).

#### Social factors

The laser radiation of ALWS can harm nearby humans and animals during its operation. This issue is especially pressing in Nordic countries, where the right to roam allows people free access to private farmland for recreation and exercise. However, human and animal safety can be ensured by several interventions (Andreasen et al., 2022). First, infrared cameras and sensors (e.g., stereovision, LIDAR, thermography sensors) can be mounted on ALWS to detect obstacles and automatically instigate control manoeuvres or shut down the system to avoid any contact with humans and animals (Reina et al., 2016). Second, operators can wear protective glasses, clothing and gloves when approaching an active ALWS. Third, laser-absorbing curtains and screens should be installed to prevent laser beams from reflecting into surrounding areas.

A further complication is that laser beams can ignite dry materials in the field during dry seasons. This could cause fires, especially when the ALWS is operating without any human supervision. Hence, ALWS units would require heat or smoke detectors in certain settings.

Dramatic progress in the development of robots and AI has allowed some non-standardised tasks that used to be reserved for human labour, such as selective weeding, to be conducted autonomously (Marinoudi et al., 2019; Young & Pierce, 2014). Furthermore, the disruption of the inflow of migrant seasonal workers into the EU due to strict COVID-19 travel measures has accelerated the adoption of robotic solutions in certain agricultural sectors (Mitaritonna & Ragot, 2020). Even though existing agricultural robots cannot completely replace human labour, they can substantially reduce the need for low-skilled human labour in the future (Marinoudi et al., 2019; Vermeulen et al., 2018). Therefore, given its highly autonomous system and advanced AI sensors, ALWS can have a negative impact on the employment rate in the long term, especially of low-skilled agricultural workers.

#### Technological factors

The experts pointed out that the efficacy of laser treatment depends on the cultivation stage. Particularly, previous studies indicated that laser treatment is most effective if applied to weed meristems in the cotyledon or two-permanent-leaf stages, when weed plants are still small (Marx et al., 2012; Mathiassen et al., 2006). Larger plants require a higher lethal weeding dose (Ascard, 1995). Increased doses might be not feasible as high-powered laser beams can split into two during the weeding process and harm crops (Rakhmatulin & Andreasen, 2020).

Despite the abovementioned drawbacks, the rapid modernisation of the agricultural sector can be beneficial for the development of sustainable practices such as ALWS (Knickel et al., 2017). For example, learnings from the operation of unmanned aerial vehicles regarding safety issues and automation design can be extended to research on ALWS (Wang et al., 2019a, 2019b). Furthermore, some of the functional components of ALWS (such as recognition systems and autonomous vehicles) have already been developed in existing machinery (Raja et al., 2019; Shaner & Beckie, 2014). Hence, these components can be inherited from or combined flexibly with other systems to accelerate the adoption process. For example, laser and recognition systems can be mounted on tractors to (1) avoid the development time required for autonomous vehicles, (2) save space in farm warehouses with fewer machines, and (3) reduce the additional cost of new machinery. In essence, the progressive development of robotic platforms in recent years (Gonzalez-De-Santos et al., 2020) can enhance both the advancement and the adoption of ALWS. However, simultaneously, the development of other physical weed-control techniques, which use microwaves, UV radiation, electrostatic fields and electrocution, can be direct market competitors for ALWS (Young & Pierce, 2014).

#### Legal factors

In the EU, no specific regulatory regime exists for the use of digital technologies in agriculture and the operation of autonomous agricultural robots. Hence, the legal framework that would be applicable to ALWS is a combination of several legal acts that relate to different fields in the laws of both the EU and of different nation states. Particularly, three main legal fields are of interest: safety, civil liability, and privacy in terms of data protection and sharing.

The EU’s product safety legislation aims to ensure that only safe products are placed on the EU’s internal market. Hence, agricultural robots, ALWS included, must meet the essential health and safety requirements laid down in the applicable EU legislation. Such legislation includes the Machinery Directive, and the directives governing the safety and health of workers at work. Concerning laser safety regulations, several regulations and standards are relevant, such as Directive 2014/35/EU on low voltage; Directive 2006/25/EC on artificial optical radiation; EN 60825-1 for laser classification and safety requirements; EN ISO 11553-1 and 11553-2 for safe machine construction; EN 60825-4 to ensure laser-safe enclosure/housing for the laser-irradiation unit; EN 60204-1 for requirements that relate to the electrical equipment of machinery; EN ISO 13849-1 and 13849-2, EN 61508-1 and EN 62061 for regulations regarding the correct choice of SRP/CS and the design and integration of safety-related parts of control systems.

ISO 19487 for agricultural machinery and tractors is not fully applicable to the autonomous vehicles that feature in ALWS. Most current production safety frameworks were written before the age of digitalisation. Hence, these legislative frameworks do not contain all the provisions that explicitly address the challenges and needs of emerging technologies. The Machinery Directive is under revision, and a new directive is being proposed to address issues that may arise from the technical progress in agriculture (CECIMO, 2021). The Organisation for Economic Co-operation and Development (OECD) is also working on its Standard Codes for the Official Testing of Agricultural and Forestry Tractors (OECD, 2022).

Civil liability legislation is also crucial in respect of ALWS. On the one hand, liability rules ensure that people who suffer harm from agricultural robots are compensated sufficiently. On the other hand, these rules provide economic incentives for the liable party to avoid causing such damage in the first place. Currently, the EU legal framework on civil liability is based on (1) the highly harmonised EU rules on the liability of the producer of a defective product (product liability directive 85/374/EEC), which covers most business-to-consumer relations; and (2) other non-harmonised national liability regimes. If an accident involving agricultural robots occurs, relations between the owners, managers, manufacturers, designers of the systems and victims should be considered. Since ALWS are autonomous, the device can make decisions without external control and influence. This feature makes it difficult to define responsibility in the case of accidents. Furthermore, ALWS is designed to work in privately owned farmland, thus the rules for self-driven vehicles are not applicable. Given the specific and new legal issues that emanate from agricultural robotic equipment, EU institutions and member states are still seeking solutions. In the interim, national laws can be applied to deal with specific cases. One of the potential solutions for liability-related challenges are to install data login systems that can help identify whether responsibility lies with the manufacturer or the user.

Agricultural robots, including ALWS, can collect valuable data on topography, production yield and other aspects of production (Wolfert et al., 2017). However, legal and regulatory frameworks that govern the collection, sharing and use of such data remain lacking. Wiseman et al. (2019) argued that the lack of transparency and clarity on data ownership, portability, privacy, trust and liability may hamper the willingness to adopt smart farming technology like ALWS. Hence, it is of critical importance to determine the ownership and governance of data generated by ALWS to avoid potential hurdles for farmers regarding data management.

#### Environmental factors

The common chemical and mechanical methods of weed control have many negative impacts on the environment (Mileusnić et al., 2022; Rani et al., 2021). Water pollution due to pesticides damages the aquatic biosystem in surface water, such as streams, lakes and ponds (Scholz et al., 2012). Silva et al. (2019) found 76 pesticide residues in 317 agricultural topsoil samples. The samples accounted for 80% of the tested soils in 16 main cropping systems in 11 EU member states. In addition, chemical pesticides have substantial negative impacts on biodiversity. Sánchez-Bayo and Wyckhuys (2019) found that chemical pollution, including pesticides, is the second biggest driver of diminished insect populations worldwide. The scientific community agrees that pesticides are one of the main factors causing the decline in terrestrial biodiversity (Brühl & Zaller, 2019). The loss in biodiversity of insects and non-target weeds can result in an insufficient food supply for higher-order animals. The residue of pesticides in the food chain can have a major effect on predators, raptors and humans as a result of bioaccumulation. Furthermore, mechanical weeding machinery that are used with heavy and large tractors can cause soil compaction (Batey, 2009). Soil tillage can disturb soil structure, reduce soil fertility and harm beneficial organisms that live on and in soil surfaces (Andreasen et al., 2022; Chatterjee & Lal, 2009).

To the best of the authors’ knowledge, the CO_2_ emissions and energy consumption of laser weeding systems have not been investigated. However, Coleman et al. (2019) indicated that site-specific weed control treatments can reduce energy use by 97–99% compared to the corresponding conventional herbicidal, thermal and machinal weed controls. As ALWS is a precision agricultural technique, it also potentially requires minimal energy consumption. Besides, based on a life-cycle assessment by Lagnelöv et al. (2021), self-driving electric tractors with batteries produced substantially less CO_2_ compared to their fossil-fuel counterparts. Hence, the choice of energy source will critically affect the impact of ALWS on global warming.

As a sustainable approach that involves no chemicals, ALWS can address the environmental problems caused by current conventional weed control practices. Since a laser beam is tiny (diameters of 2–3 mm), the area treated by ALWS is small. For example, if the laser beam has a diameter of 3 mm and 100 weed plants/m^2^ need to be controlled, the exposed area is equal to 1.5^2^ × π = 710 mm^2^, which is 0.71% of the total area. Furthermore, the experts who contributed to this study expected an ALWS to be lighter than common mechanical weeders. Hence, the negative impact of laser treatment on surrounding organisms is significantly less than that of mechanical weeders.

Undesirable weather, such as heavy rains or drought, can complicate the operation of ALWS. During rainy periods, the ground may become too slippery for AWLS movement. In this regard, Lucet et al. (2015) introduced a path-tracking control for field robots, which helped the robots in their study speed up to 7 m/s in terrain of wet grass. Such an advanced kinematic model may be required to assure the movement stabilisation of ALWS in adverse weather conditions. Moreover, agricultural vehicles are more likely to cause soil compaction in wet soil (Ren et al., 2019), hence designing ALWS as a light vehicle is desirable to assure its efficient and optimal operation in rainy periods.

A further weather-related challenge is that water drops can redirect laser beams and/or protect the weeds from the beam. Lightning can also harm the vehicle in the field. In addition, dry fields with inflammable materials, such as straw and dried leaves, can easily be set on fire by the laser beams. Consequently, smoke sensors and surveillance of ALWS and the treated area should be considered to avoid fire risks under certain conditions (Andreasen et al., 2022).

Farmland with obstacles such as stones, power poles and water lines can impede the movement of ALWS. However, the latest technological advances and the fusion of different sensor technologies are allowing safer navigation in the field (Reina et al., 2016). Nevertheless, vibration caused by movement on uneven surfaces can divert laser beams to hit crop plants, thus reducing the efficacy of ALWS. Proper seedbed preparation may therefore be essential to ensure the optimal function of ALWS (Andreasen et al., 2022).

##### SWOT analysis

**Fig. 3:**
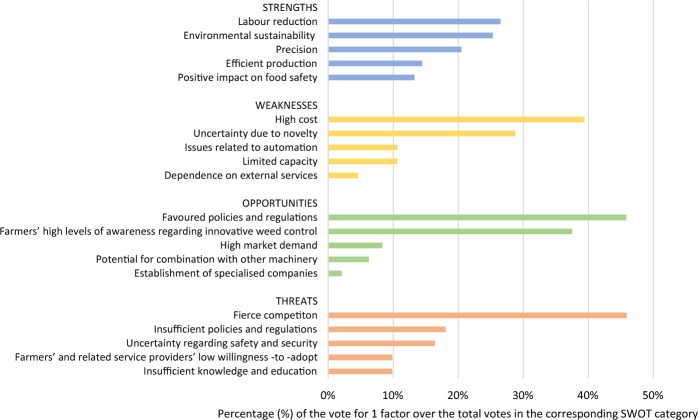
Important SWOT factors* for the adoption of ALWS from stakeholders’ perspective (n = 55). *Factors were considered important if they were among the top five voted for by the focus-group participants. Factors that received few votes are not displayed

Figure 3 illustrates the SWOT factors that stakeholders regarded as important for the adoption of ALWS. This section presents the results of the SWOT analysis of the contributions made by the four focus groups. These empirical results were compared with the extant literature of stakeholders’ perceptions of precision agriculture adoption.

#### Strengths

*Labour reduction* The stakeholders stressed that the shortage of agricultural labour in Europe and the consequent high cost of low-skilled labour make autonomous systems such as ALWS more desirable to farmers. Similarly, in a study by von Veltheim and Heise (2020), German farmers stated that the trend to reduce pesticide use has necessitated more labour-intensive weed control—a burden that autonomous field robots can alleviate. A similar trend is experienced in US: Carolan (2020) reported that US farmers considered agricultural robots as a long-term solution to labour scarcity and the high cost of labour.

*Environmental sustainability* The focus-group participants regarded environmental sustainability as the main advantage of ALWS. Given its precision and the resultant reduction in soil disturbance, ALWS has a greatly reduced impact on living creatures and surrounding areas, thus protecting biodiversity. Furthermore, ALWS eliminates herbicide use from weed control, thereby reducing dependence on phytosanitary products and the emergence of herbicide-resistant weeds. According to the stakeholders, ALWS machinery is likely to be less heavy than conventional mechanical weeders, thus reducing the risk of soil compaction.

*Precision* Because ALWS is assisted by a recognition system, it can target in-row weeds and avoid damage to crops. According to the stakeholders, the recognition system and vehicle component of ALWS allow it to identify and eradicate weeds flexibly and precisely, which in turn minimises the dependence of ALWS on uniform crop rows. This feature is particularly useful in the case of intercropping, where row structures may vary from crop to crop.

*Efficient agricultural production* The stakeholders viewed ALWS as a promisingly efficient production method. By using remote control and supervision systems, one operator can manage several robots working in the field simultaneously. Theoretically, ALWS can work 24/7, maximising the time used and offering farmers greater flexibility, thus easing their concerns about weed control.

*Positive impact on food safety* The use of ALWS can improve food safety because this method generates no chemical residues in food products. In addition, a waiting period after herbicides have been applied is no longer necessary.

#### Weaknesses

*High cost* Even though ALWS is not yet commercially available in Europe, the stakeholders anticipated that the high price would be the greatest drawback. Nearly 40% of the participants voted for this factor. Swiss experts in a Delphi study also considered cost as the most critical factor in the adoption of new technology for outdoor vegetable production (Ammann et al., 2022). Furthermore, the maintenance and operation costs of ALWS were expected to contribute to high operational costs. Generally, these findings were not unexpected as economic costs have been widely acknowledged as one of the main barriers to the adoption of new technologies in agriculture (Barrett & Rose, 2022; Hashem et al., 2021). In this regard, Barnes et al. (2019) suggested that providing farmers with an estimation of the viable economic return on the technology can encourage farmers to overcome their concern over costs.

*Uncertainty due to novelty* Some stakeholders were concerned that novel techniques like ALWS might not provide sufficient evidence of their effectiveness. Most ALWS have demonstrated optimal conditions only in laboratories or designated fields. Diverse real-life conditions, such as undesirable weather and uneven surfaces, may hamper the performance of ALWS. Furthermore, the indistinctive morphology of certain weed types may mean that the weed recognition system may require more time to learn and adapt to local situations. These concerns showed that stakeholders lacked confidence in such a novel technology as ALWS. In this regard, Michels et al. (2021) found that farmers’ high levels of confidence in technology are significantly associated with a high intention to adopt. To gain farmers’ trust in new technologies, including ALWS, it is recommended that farmers should be provided with realistic and easily understandable performance results achieved by the machinery.

*Limited capacity* Based on the stakeholders’ experience, the capacity of treatment (hectare per day) of agricultural robots like ALWS is often limited, even when working 24/7 because the recognition systems take time to perform. This concern in respect of precision weeding is also well documented in the literature (Pedersen et al., 2006). Weed control is intensive only at certain times of the year. Hence, the slow speed and the small surface area that ALWS is able to cover can mean that several ALWS would be required in a field at a given time. This practice might not be economically viable for farmers.

*Issues related to automation* Some stakeholders stated that the automation features can be viewed as a shortcoming for several reasons. First, an autonomous vehicle is vulnerable to theft and sabotage by competitors because human supervision is absent. Second, incidents of malfunction in the field may not be sufficiently monitored and timeously avoided, for example, when the laser beams ignite a fire. Third, current regulations regarding autonomous systems require certain security measures to ensure human safety and liability for damage to property (Spykman et al., 2021). According to the stakeholders who participated in this study, such additional requirements may mean more costs for farmers. Fourth, for the purposes of navigation and remote control, an autonomous system may rely on a global navigation satellite system (GNSS) and/or an internet connection (Tzounis et al., 2017). However, many farms remain outside the range of 4G technology, even in developed countries (Tang et al., 2021; USDA, 2019). The autonomous system might therefore struggle to function efficiently without a stable internet connection in remote areas.

*Dependence on external services* Some stakeholders stated that ALWS is a sophisticated system that may require specialised technical services for maintenance and operation. For example, the transport of ALWS between farms may need to be performed by a container truck. These additional prerequisites can discourage farmers from investing in ALWS. Similar to our findings, more than 60% of Bavarian farmers surveyed also considered the increased dependence on the providers of such machinery as an obstacle to the adoption of field crop robots (Spykman et al., 2021).

#### Opportunities

*Favoured policies and regulations* The increasingly stringent policies and legislation governing chemical weed control promote the adoption of sustainable alternatives. As discussed in the PESTLE analysis, ALWS aligns with the long-term vision and legal framework of (increasingly organic) agricultural production in Europe.

*Farmers’ high levels of awareness regarding innovative weed control* Rapid agricultural modernisation is beneficial for the development and adoption of ALWS. According to the stakeholders, farmers’ awareness of innovative techniques has been increasing in recent decades, paving the way for the adoption of tools like ALWS. This opinion is in line with the findings of Skevas et al. (2022) that awareness of new technology (in their case, unmanned aerial drones) significantly impacted on adoption by American farmers.

*High market demand* Some stakeholders stated that conventional farmers can improve their sustainability by taking advantage of the move towards and support for innovative organic methods. The expansion of organic farming has seen increased investment in innovative techniques such as ALWS (Ulmann, 2020). According to the stakeholders who participated in this study, the early adoption of ALWS can provide a competitive advantage as this technique may outperform current weeding methods in the long run.

*Potential for combination with other machinery* Some farmers mentioned that ALWS can be combined with their existing precision agricultural machinery. Precision weed control techniques, such as ALWS, can detect and kill in-row weeds. However, since weed recognition is time-consuming, the operation speed of ALWS (around 1–2 km/h) is significantly slower than that of common mechanical weeders (around 4–6 km/h). Furthermore, common mechanical weeders adequately eliminate inter-row weeds, but they cannot target in-row weeds (Rabier et al., 2017). For some crops, such as sugar beet, using only mechanical weeders is not enough to ensure a decent yield because in-row weeds become dominant (Rabier et al., 2017). The combination of mechanical weeders and ALWS would therefore be of interest if the operation speed of ALWS could be improved and if ALWS was used after mechanical weeders had already eradicated inter-row weeds. In addition, it is possible for ALWS to be integrated with other existing robotics systems, such as field mapping robots (Slaughter et al., 2008) to maximise the efficiency of the current systems (e.g., irrigation, fertilisation) and accelerate the adoption process.

*Establishment of specialised companies in agricultural services* Some of the stakeholders in the focus groups indicated that the recent establishment of specialised companies in agricultural services helps to address the need for additional services that precision agricultural machinery, such as ALWS, requires. Precision machinery often needs to be maintained by specialised technicians, and trained operators might even be needed for operation in the field. Hence, the unavailability of service support can hamstring farmers’ access to innovative farming techniques (Silvi et al., 2021). Participants proposed that farmers can periodically rent agricultural machinery from service providers at a reasonable price. This approach would be cost effective for both farmers and machinery providers as (1) agriculture production is seasonal, and (2) allowing machines to be idle for extended periods can cause further maintenance expenses.

#### Threats

*Fierce competition* Some machinery developers and dealers were concerned that the long development process of ALWS threatens the competitiveness of this application. Chemical and mechanical weed control has advanced, offering precision systems that meet stringent regulations and market demands. For instance, precision sprayers for site-specific weed management are now common on many large-scale farms, though these systems use chemicals (Späti et al., 2022). The existence of physical solutions as alternatives to chemical weed control intensifies the fierce competition facing ALWS. At present, it is unclear whether the commercial model of ALWS can compete with existing organic-farming solutions in terms of profitability and technical performance. Hence, providing an investment analysis that would demonstrate the viable economic returns of ALWS might help convince farmers of the potential of ALWS.

*Insufficient policies and regulations* Despite the abovementioned policies that favour the adoption of ALWS, certain existing regulations can be considered obstacles to the adoption of ALWS. For example, green-energy policies may hamper the use of a combustion engine in ALWS. If the strict legislation applicable to autonomous machines is not adequately adapted for agricultural machinery, the legal requirements would deter the widespread adoption of ALWS. Participants also mentioned that the lack of incentives, such as direct payment, to early adopters can delay the adoption. This lack of incentives also applies to other precision agricultural techniques: As Späti et al. (2022) argued, welfare measures can significantly stimulate the uptake of site-specific nitrogen fertilisation in Switzerland. However, the stakeholders who participated in this study raised the concern that even if subsidies for early adopters were available, beneficiaries could struggle to access such support because of the complicated bureaucratic procedures involved.

*Uncertainty regarding safety and security* Stakeholders expressed concern over the vulnerability of ALWS to theft and vandalism because the system operates with limited human supervision. Furthermore, ALWS has the capability to collect and store sensitive data regarding field mapping and production, making such systems vulnerable to cyberattacks. In the study of von Veltheim et al. (2022), German farmers agreed that data protection plays an important role in the adoption of autonomous field robots. Likewise, Australian farmers stated that they lacked trust in the way their farm data is being collected and managed (Wiseman et al., 2019). Moreover, determining liability is a challenge because current regulation does not stipulate specific terms for agricultural robots in the case of accidents.

*Insufficient knowledge and education of farmers* Stakeholders mentioned that formal education in agronomy cannot keep up with the rapid development of agricultural machinery. This view is supported in the extant literature, which found that farmers’ limited education and knowledge hinder the adoption of smart farming (Michels et al., 2020; Pivoto et al., 2018). Some stakeholders suggested that machinery interfaces should be more user-friendly, making it easier for farmers to use their machines. Nevertheless, farmers need to be trained in precision techniques to promote the widespread adoption of ALWS (Barrett & Rose, 2022; Redhead et al., 2015). In the interim, suitable promotion (e.g., field demonstrations and agricultural machinery trade fairs) can bring innovation to the attention of farmers’ attention. Ultimately, such exposure would create greater interest in such technology.

*Farmers’ and related service providers’ low willingness to adopt* Given the previously mentioned drawback of ALWS, farmers may hesitate to adopt such a novel technique. In fact, a survey of German farmers by Spykman et al. (2021) found a relatively low rate of intention to adopt field robots (22%). Participants in this study also stated that to adopt innovative machinery, farmers require clear facts regarding the machine’s performance, which new techniques may be unable to provide. Furthermore, companies that provide only agricultural machinery and the servicing of conventional mechanical weeders may be unwilling to add new and innovative products to their portfolio because of concerns over low profitability in the early stages. In this regard, Spykman et al. (2021) emphasised that the unavailability of robotic machinery and market immaturity are two of the critical challenges to the adoption of field robots by farmers.

### Combined findings of PESTLE and SWOT analysis

Table 3 illustrates the merged findings of the PESTLE and SWOT analyses: PESTLE factors were classified into SWOT categories and vice versa. This mixed method provides a comprehensive view of the adoption potential of ALWS.

**Table 3:** Combined PESTLE and SWOT analysis

	Strengths	Weaknesses	Opportunities	Threats
Politics			**Favoured policies (Green Deal, CAP)**	**Insufficient policies (e.g., farmers’ incentives)**
Economics	**Labour reduction**	**High cost**	**High demand for organically farmed products** **High agricultural labour cost**	***Crises (COVID-19, energy)*** **Fierce competition**
Society	*Positive impact on food safety*	***Lower the rate of low-skilled employment***	*Farmers’ high levels of awareness regarding innovative weed control* *Establishment of specialised companies*	**Human safety (users, right to roam)** *Insufficient knowledge and farmers’ education* *Farmers’ and related service providers’ low willingness-to-adopt*
Technology	**Precision (in-row weeding)** *Efficient production (24/7)*	***Short (optimal) treatment period*** *Uncertainty due to novelty* *Limited capacity* *Automation issues (e.g., low connection in remote areas)* *Dependence on external services*	**Potential for combination with other machinery**	**Theft, vandalism** **Laser igniting fire** **Data security**
Legislation			**Favoured regulations (e.g., stricter chemical use)**	**Insufficient regulations (e.g., lack of regulation for argi-robots, farming data)**
Environment	**Organic farming (no chemicals)** **Less soil compaction and disturbance** **Preserve biodiversity**			**Undesirable weather** **Uneven farmland**

Bolditalic factors were indicated in the PESTLE analysis only, italic factors were indicated in the SWOT analysis only, and bold factors were indicated in both the PESTLE and SWOT analyses

Most of the factors were identified in both analyses. Technical factors (mostly identified in the SWOT analysis), such as the efficiency and capacity of the ALWS prototype compared to conventional solutions, and the positive impact on food safety, can be tested empirically. Similarly, stakeholders’ speculative opinions about farmers’ levels of awareness, knowledge and willingness to adopt can be confirmed by taking a quantitative approach and conducting surveys.

The issue of the short period in which ALWS can be used optimally was identified in the PESTLE analysis only. This study recommends that the implications should be transparently communicated to all stakeholders. This would avoid disappointment and farmers’ consequent distrust in the new technology. The negative impact of ALWS on the rate of low-skilled employment was considered in the literature only. Participants in this study did not vote for this issue as one of the most-significant factors affecting the adoption of ALWS. The reason might be that low-skilled agricultural workers were not included in this study’s sample population. Besides, crises such as the shortage in energy and COVID-19 were not highlighted by the participants. This suggests that their views on the adoption of ALWS might be constrained by a microeconomic-centric view.

### Limitations

The SWOT analysis was based on the perceptions and knowledge of a limited number of stakeholders. The findings may therefore reflect personal bias, which is common in explorative studies (Bitsch, 2005). Nevertheless, the number of stakeholders (n = 55) and the diversity of their backgrounds (farmers, machinery producers/providers, researchers and policymakers) strengthens the validity of this study’s findings (Olum et al., 2018).

The four focus groups met separately, and some of the SWOT factors were not displayed to all the stakeholders for voting. This discrepancy should be considered when interpreting the results of this study. A two-round Delphi study could rectify this by collecting all the factors put forward by the stakeholders in the first series of workshops. Participants could then vote for a complete list of factors by means of a questionnaire or in a second round of workshops. This avenue might be considered for future research (Campos-Climent et al., 2012).

The purpose of the voting procedure that formed part of the SWOT analysis was for stakeholders to evaluate the factors that they had identified. To enhance the robustness of the SWOT findings, more advanced methods, such as an analytical hierarchy process (AHP; Olum et al., 2018) or strategic orientation round (SOR; Rutsaert et al., 2014), can be conducted to quantitatively appraise the importance of the factors.

Furthermore, because this study focused on Europe, its findings may not apply to agricultural machinery sectors in other regions. The levels of advancement in agricultural technologies, production cost structures and related policies may differ significantly. However, because the achievement of sustainable agricultural practices is a goal internationally, this study’s findings are relevant and contribute to our understanding of the potential of ALWS and other similar precision technologies, at least in the European market and other developed countries in the foreseeable future. Furthermore, although this study involved stakeholders from several European countries, future research could complement and/or confirm our findings with a larger, more representative sample of stakeholders from Europe and other regions.

### Summary and conclusions

This study combined the findings of PESTLE and SWOT analyses to provide a comprehensive overview of the adoption potential of ALWS. To be specific, a PESTLE assessment identified the most important macroenvironmental factors that affect the adoption of ALWS. These factors shaped the literature review. In addition, a SWOT analysis explored stakeholders’ perceptions of ALWS.

The European stakeholders who participated in this study were found to have a positive attitude to ALWS because this solution addresses the challenges posed by labour shortages and the negative environmental impact of conventional weed-control solutions (damaged biodiversity, soil disturbance and compaction, and CO_2_ emissions). In addition, the precision of ALWS allows greater flexibility in crop cultivation, while its autonomy and ability to work 24/7 optimises production time. Such a system also eliminates the need for herbicides, thus reducing the risk of harmful chemical residues in food.

Participants viewed high implementation costs as the major weakness. Conducting an investment analysis is essential to convince farmers of the potential of ALWS. Stakeholders were also uncertain of the performance of ALWS: They perceived ALWS to have limited capacity and foresaw issues with autonomous function and a dependence on external services. An autonomous solution without supervision sparked particular concerns about human safety, machine safety (e.g., theft, vandalism, fire risks) and liability regulations. Furthermore, integrating an autonomous component may slow down the introduction of a laser weeding solution as a whole. Developers can consider mounting laser components on tractors as an alternative to autonomous vehicles. Regardless of the mobility approaches, technical factors and their implications in field operation should be transparently communicated with farmers so they can make informed decisions and have trust in the new solution. These marketing communications can be provided in field demonstrations, trade fairs, or via connection with farmers’ organisations and cooperatives.

The current business environment in Europe seems promising for the introduction of ALWS. Policies that favour the adoption of sustainable agriculture and the stringent regulation of herbicide use position ALWS as a sustainable substitute for conventional solutions. Similarly, the booming market for organic products is a bright prospect for the adoption of ALWS because this technique eliminates the need for manual weeding, which incurs the highest proportional cost in organic production. However, fierce competition in the European machinery market necessitates the accelerated development of ALWS.

The implementation of new technologies, such as ALWS, cannot proceed smoothly without an adequate legal framework to govern its application. Hence, an urgent call is made for legislators to attend to insufficient regulations regarding the civil liability of agricultural robotic equipment and the protection of farming data.

Lastly, policymakers are advised to consider the timely provision of training in precision agriculture to farmers. In addition, advisory support should be available to improve farmers’ understanding of the new technology and assist them in the implementation process. Furthermore, financial support (e.g., direct payment for sustainable practices and tax reduction) that is easy to access is needed to overcome the cost barriers facing farmers. In conclusion, effective policy measures, clear communication with farmers, and robust technological advancements in precision agriculture could pave the way for ALWS to become a game-changer for weed control in Europe.
